# Surgical Outcomes in Laparoscopic Hysterectomy, Robotic-Assisted, and Laparoscopic-Assisted Vaginal Hysterectomy for Uterine and Cervical Cancers: A Systematic Review

**DOI:** 10.3390/diagnostics14242782

**Published:** 2024-12-11

**Authors:** Jabri Tabrizi (Plic) Madalina Ioana, Florica Voiță-Mekereș, Alexandru Catalin Motofelea, Duta Ciprian, Lazăr Fulger, Isaic Alexandru, Cristi Tarta, Pantea Stelian, Elena Silvia Bernad, Hoinoiu Teodora

**Affiliations:** 1 Doctoral School, “Victor Babes” University of Medicine and Pharmacy Timisoara, 300041 Timisoara, Romania; madalina.plic@umft.ro; 2 Center for Advanced Research in Cardiovascular Pathology and Hemostaseology, “Victor Babes” University of Medicine and Pharmacy Timisoara, 300041 Timisoara, Romania; tstoichitoiu@umft.ro; 3 Department of Morphological Disciplines, Faculty of Medicine and Pharmacy, University of Oradea, 410087 Oradea, Romania; 4 Department of Internal Medicine, “Victor Babes” University of Medicine and Pharmacy Timisoara, 300041 Timisoara, Romania; alexandru.motofelea@umft.ro; 5 Department of General Surgery, “Victor Babes” University of Medicine and Pharmacy Timisoara, 300041 Timisoara, Romania; dutaciprian@umft.ro (D.C.); lazarfulger@umft.ro (L.F.); isaic.alexandru@umft.ro (I.A.); tarta.cristi@umft.ro (C.T.); pantea.stelian@umft.ro (P.S.); 6 Department of Obstetrics and Gynecology, Faculty of Medicine, “Victor Babes” University of Medicine and Pharmacy Timisoara, 300041 Timisoara, Romania; bernad.elena@umft.ro; 7 Department of Clinical Practical Skills, “Victor Babes” University of Medicine and Pharmacy Timisoara, 300041 Timisoara, Romania

**Keywords:** laparoscopic hysterectomy, robotic-assisted hysterectomy, laparoscopic-assisted vaginal hysterectomy, gynecological cancers, surgical outcomes, minimally invasive surgery, systematic review

## Abstract

Background/Objectives: This systematic review aimed to evaluate the outcomes of minimally invasive techniques in gynecological cancer surgery, specifically laparoscopic hysterectomies (LHs), robotic-assisted hysterectomies (RHs), and laparoscopic-assisted vaginal hysterectomies (LAVHs). Methods: We conducted a comprehensive search of electronic databases including PubMed and MedLine from January 2010 to August 2024. The search included randomized controlled trials (RCTs) and observational studies. Studies were selected based on inclusion criteria such as a focus on LHs, RHs, or LAVHs, and reporting on key outcomes like recovery rates, overall survival (OS) rates, disease-free survival (DFS), postoperative complications, and surgery time. Exclusion criteria were applied to omit non-randomized studies, non-English publications, and those lacking relevant data. Results: The analysis included 35 studies on gynecological cancers and surgical procedures, conducted across multiple countries. Among them, 8 were RCTs from countries like the Netherlands and Italy, while 20 were retrospective cohort studies from China and the USA. The studies varied in design, cancer type, and participant age, highlighting diverse surgical approaches and the adaptation of minimally invasive techniques in gynecological cancer treatment. LH and RH demonstrated similar oncological safety with comparable OS and DFS rates. RH was associated with reduced blood loss, but longer operative times compared to LH. LAVH showed favorable perioperative outcomes, including shorter hospital stays and faster recovery, but was less frequently studied in advanced-stage cancers. Complication rates were generally lower in minimally invasive surgeries compared to open procedures. The findings support the efficacy of LH and RH as viable alternatives to open surgery, with specific advantages depending on patient and disease characteristics. Conclusions: Minimally invasive techniques in gynecological cancer surgery offer significant advantages in terms of recovery and complication rates. Despite these benefits, further research is needed to confirm their oncological safety and overall effectiveness compared to traditional open surgeries.

## 1. Introduction

Gynecological cancers, including cervical and uterine malignancies, represent a major global health issue, impacting millions of women each year. According to Yuanhao et al., in 2019, there were approximately 400,146 new cases of premenopausal and 879,476 new cases of postmenopausal gynecological cancers worldwide. The study also highlighted 111,420 deaths in premenopausal women and 442,821 deaths in postmenopausal women. Projections suggest an increase in premenopausal gynecological cancer cases by 2040, whereas postmenopausal cases are expected to decline [1].

Endometrial cancer, the most common of these gynecological malignancies, predominantly affects women in high-income countries. The Global Cancer Observatory (GLOBOCAN) reported approximately 417,000 new cases of endometrial cancer in 2020, making it the sixth most prevalent cancer among women globally. This same year, endometrial cancer was responsible for about 97,000 deaths, highlighting its significant impact on women’s health [2,3].

Cervical cancer remains a leading cause of cancer-related mortality among women, especially in low- and middle-income countries, despite advances in screening and vaccination. GLOBOCAN data from 2020 revealed over 604,127 new cervical cancer cases worldwide, with a corresponding 341,831 deaths. The high mortality rate is largely attributed to late-stage diagnoses and limited access to effective treatment, underscoring the need for enhanced prevention, early detection, and surgical interventions [4,5].

Uterine cancer, encompassing endometrial cancer as well as rarer types of uterine malignancies, also represents a significant part of the gynecological cancer burden. Although they are less common than endometrial cancer, uterine sarcomas and other non-endometrioid tumors are often more aggressive and associated with poorer outcomes [6,7].

Surgical management of these cancers has advanced significantly, with minimally invasive techniques now preferred over traditional open surgeries. Laparoscopic hysterectomy (LH), robotic-assisted hysterectomy (RH), and laparoscopic-assisted vaginal hysterectomy (LAVH) are among the most commonly used minimally invasive options [8]. These techniques offer several advantages, including reduced perioperative morbidity, shorter hospital stays, faster recovery times, and lower complication rates compared to open surgery [9].

Despite these benefits, there are ongoing questions about the relative efficacy and safety of these minimally invasive techniques, especially concerning oncological outcomes such as overall survival (OS) and disease-free survival (DFS). Additionally, the impact on perioperative factors such as estimated blood loss (EBL), operative time, and postoperative recovery remains crucial for both clinical decision-making and patient outcomes [9,10].

This systematic review aims to critically evaluate and compare the surgical outcomes of LH, RH, and LAVH for the treatment of endometrial, cervical, and uterine cancers. By synthesizing the available evidence, this review seeks to provide a comprehensive assessment of the benefits and limitations of each technique, thereby informing clinical practice and improving patient outcomes in the surgical management of gynecological cancers.

## 2. Materials and Methods

### 2.1. Study Design

This systematic review was conducted to evaluate and compare the surgical outcomes of three minimally invasive hysterectomy techniques—LH, RH, and LAVH—in the treatment of gynecological cancers. The review followed the Preferred Reporting Items for Systematic Reviews and Meta-Analyses (PRISMA) guidelines, ensuring a structured and transparent approach to the identification, selection, and analysis of relevant studies [11]. This review was registered in the PROSPERO International Prospective Register of Systematic Reviews under the registration number CRD42024594418.

### 2.2. Search Strategy

A comprehensive electronic search was performed across multiple databases, including PubMed and Google Scholar. The search was restricted to articles published between January 2010 and June 2024 to ensure the inclusion of recent advancements in minimally invasive surgical techniques. The following keywords and MeSH terms were used in various combinations: “laparoscopic hysterectomy”, “robotic-assisted hysterectomy”, “laparoscopic-assisted vaginal hysterectomy”, “gynecological cancers”, “surgical outcomes”, “endometrial cancer”, “ovarian cancer”, “cervical cancer”, “perioperative outcomes”, “complications”, “disease-free survival”, and “overall survival”. Additional manual searches were conducted by screening the reference lists of identified articles to capture any studies that may have been missed during the initial database search.

### 2.3. Eligibility Criteria

Studies were included if they met the following criteria: they were (1) original research articles, including randomized controlled trials (RCTs), observational studies, and retrospective analyses; (2) studies focusing on the surgical outcomes of LH, RH, or LAVH in patients with endometrial, uterine, or cervical cancer; (3) studies reporting relevant surgical outcomes, such as perioperative morbidity, EBL, operative time, hospital stay duration, OS, and DFS; and (4) studies published in English. Exclusion criteria includeed (1) studies focusing on benign gynecological conditions, (2) studies without comparative data between LH, RH, and LAVH, (3) case reports, review articles, and editorials, and (4) studies with insufficient data for outcome analysis.

### 2.4. Data Extraction and Quality Assessment

**Figure 1:**
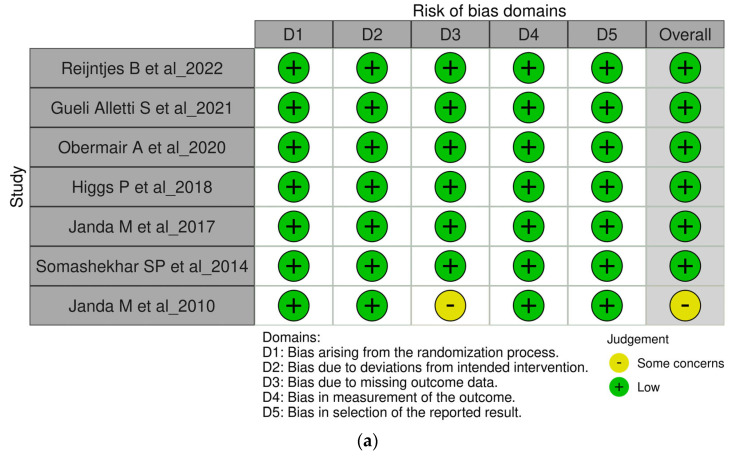

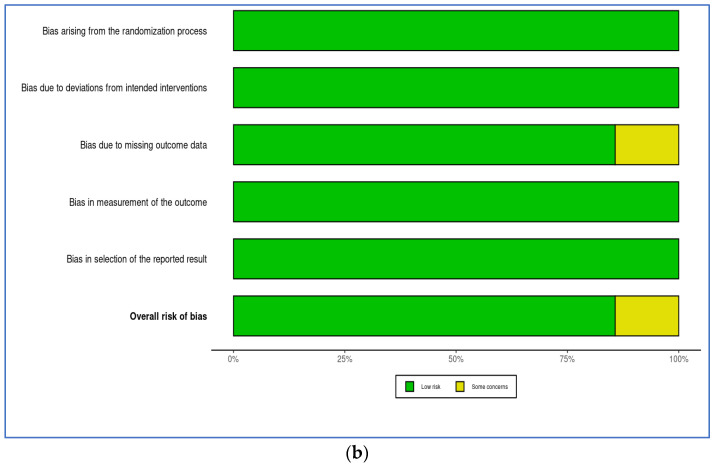
(**a**,**b**) Risk of bias assessment result for RCTs [13,14,15,16,17,18,19].

Two independent reviewers conducted the data extraction using a standardized form. Extracted data included study characteristics (author, year of publication, country, study design, sample size), patient demographics (age, cancer type, cancer stage), and reported surgical outcomes (EBL, operative time, hospital stay, OS, DFS, and complications). Discrepancies between reviewers were resolved through discussion or by consulting a third reviewer. Quality assessment was conducted using the Newcastle–Ottawa Scale (NOS) for observational studies and the Cochrane Risk of Bias Tool for RCTs [12]. Studies were rated as high, unclear, or low quality based on five ROB domains (Figure 1).

### 2.5. Data Synthesis and Analysis

A qualitative synthesis was performed due to heterogeneity in the study designs, patient populations, and outcome measures across the included studies. Descriptive statistics were used to summarize the key findings. Where possible, a narrative comparison of surgical outcomes between LH, RH, and LAVH was provided. Meta-analysis was not conducted due to the variability in the study methodologies and reported outcomes.

### 2.6. Ethical Considerations

As this study was a systematic review of the previously published literature, ethical approval and informed consent were not required. However, all included studies were screened for ethical approval and patient consent as part of their original research protocols.

## 3. Results

**Figure 2:**
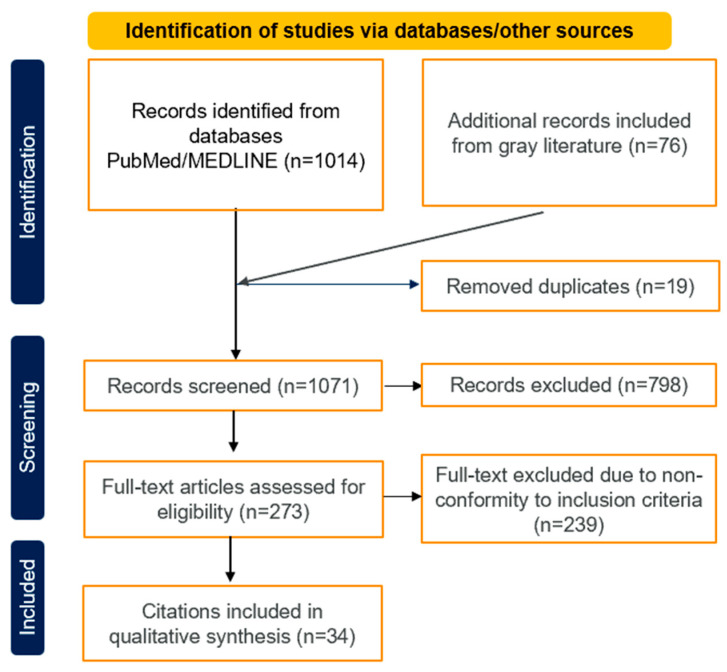
PRISMA checklist.

A total of 1014 records were identified from PubMed/MEDLINE, with an additional 76 records sourced from the gray literature. Of these, 273 full-text articles were assessed for eligibility, ultimately leading to the inclusion of 34 citations in the qualitative synthesis of the study (Figure 2).

### 3.1. Overview of Included Studies and Surgical Approaches

The analysis included 34 studies examining various gynecological cancers and surgical procedures [13,14,15,16,17,18,19,20,21,22,23,24,25,26,27,28,29,30,31,32,33,34,35,36,37,38,39,40,41,42,43,44,45,46] (Table 1). These studies were conducted in multiple countries, reflecting a broad international scope. Among the studies, eight were RCTs conducted in countries including the Netherlands, Italy, Australia, and India, as well as a multicenter study involving 24 countries. The majority were retrospective cohort studies (20) conducted in countries like China, Germany, Japan, the United States, and others. Additionally, there were three case–control studies, four prospective cohort studies, and one retrospective case–control study, highlighting diverse study designs.

Patient populations varied by cancer type and stage, with sample sizes ranging from 27 to 157,232 participants. The mean age of participants also varied, with some studies reporting mean ages as low as 20.9 years for early-stage cervical cancer and up to 80 years for older patients undergoing minimally invasive hysterectomies for uterine cancer. The most frequently studied cancers were endometrial (22 studies), cervical (7 studies), and uterine (4 studies).

Surgical approaches were diverse, including total laparoscopic hysterectomies (TLHs), total abdominal hysterectomies (TAHs), RAHs, and laparotomies. A significant portion of the studies compared minimally invasive surgery to open surgery, underscoring the ongoing evolution in surgical techniques. For example, Reijntjes et al. (2022) in the Netherlands compared TLH and TAH in stage I endometrial cancer, while Gueli Alletti et al. (2021) in Italy evaluated minimally invasive staging in early-stage endometrial cancer [13,14]. Larger studies, such as that of Yuk et al. (2021) [30] from Korea, involved 157,232 women with an unsuspected uterine malignancy post-hysterectomy, and Hayek et al. (2022) [24] in the United States analyzed 6230 women with stage IA1/IA2 cervical cancer, comparing open and minimally invasive surgical approaches. Overall, the analysis underscores the diversity in surgical approaches and highlights the adaptation of minimally invasive techniques in treating gynecological cancers.

### 3.2. Uterine Malignancies

#### 3.2.1. Summary of Surgical Approaches for Uterine Cancer

Studies on uterine cancer highlight surgical outcomes across robotic-assisted, laparoscopic, and open surgeries, focusing on OS, DFS, operative time, EBL, discharge time, and complications. Versluis et al. (2018) demonstrated that lymph node dissection (LND) with more than 10 nodes removed significantly improved survival, with OS at 2.03 years and DFS at 1.53 years [35]. For instance, in a study focused on patients aged 80 years and older with uterine cancer, Zakhari et al. (2016) [40] found that robotic surgery was associated with shorter hospital stays and a lower composite rate of postoperative complications compared to laparoscopic approaches. This evidence suggests that even among advanced-age cohorts, minimally invasive strategies can improve perioperative safety and enhance postoperative recovery. These findings suggest that robotic-assisted and laparoscopic surgeries may offer reduced blood loss and shorter hospital stays, making them preferable for many patients, with surgical approaches individualized based on patient-specific factors.

#### 3.2.2. Summary of Surgical Approaches for Endometrial Cancer

The reviewed studies offer a comprehensive analysis of surgical approaches for endometrial cancer, focusing on OS, DFS, recurrence rates, operative time, blood loss, discharge times, and complications.

- OS, DFS, and recurrence rates

Reijntjes et al. (2022) found that TLHs had a higher 5-year OS rate of 89.2% compared to 82.8% for TAHs [13]. Shuai et al. (2024) reported comparable 5-year OS rates between laparotomies and laparoscopies (85.5% vs. 82.7%) [20]. Similarly, Yuk et al. (2021) observed significantly better OS in the laparoscopic group versus the laparotomic group [30]. Jørgensen et al. (2019) highlighted that MIS, including robotic MIS (RMIS), was associated with lower mortality compared to TAH [32]. Corrado et al. (2018) noted a slight survival advantage for RHs over LHs [33]. For DFS, Reijntjes et al. (2022) reported 5-year rates of 90.3% for TLHs and 84.1% for TAHs [13]. Shuai et al. (2024) found similar DFS rates between laparotomies and laparoscopies (88.7% vs. 87.1%) [20]. Segarra-Vidal et al. (2021) observed comparable DFS rates between open surgery and MIS [26]. These findings suggest MIS approaches like TLH and RH offer a slight survival advantage with similar DFS compared to more invasive methods.

Reijntjes et al. (2022) reported a lower recurrence hazard ratio (HR) for TLHs compared to TAHs, indicating reduced recurrence [13]. However, Shuai et al. (2024) found no significant differences in recurrence rates between laparoscopic and laparotomic methods, with an overall rate of 14.3% [20]. Renaud et al. (2022) noted higher recurrence rates with laparotomy compared to MIS [27]. Janda et al. (2017) found similar recurrence rates for TAHs and TLHs [17] (Table 2).

- Operative time, blood loss, discharge time, and complications

Gueli Alletti et al. (2021) reported operative times of 150–370 min for laparoscopic and robotic surgeries, with EBL between 50 and 550 mL [14]. Renaud et al. (2022) noted shorter operative times for laparotomy (137 min) [27]. Corrado et al. (2018) observed longer operative times and slightly higher EBL for robotic hysterectomy compared to laparoscopic hysterectomy [33]. Discharge times were generally short, with a median of 2 days for MIS procedures [14]. Jørgensen et al. (2019) found more postoperative complications with TAH compared to MIS [32]. Mereu et al. (2020) reported low complication rates [31]. These results highlight the benefits of robotic and laparoscopic surgeries in terms of EBL and discharge times, despite longer operative times.

#### 3.2.3. Summary of Surgical Approaches for Cervical Cancer

Table 1 and Table 3 present a comprehensive analysis of surgical approaches for cervical cancer, focusing on OS, DFS, recurrence rates, operative time, EBL, discharge times, and complications.

- OS, DFS, and recurrence rates

Kondo et al. (2022) and Hayek et al. (2022) found no significant difference in OS between TLRH and open radical hysterectomy (O-RH) or between MIS and open surgery, with OS rates of 97.2% to 100% [22,24]. Wojdat et al. (2022) reported high 3- and 5-year survival rates in early-stage cervical cancer [25]. Nasioudis et al. (2021) also found similar OS rates between open and MIS hysterectomy groups [28]. For DFS, while Wojdat et al. (2022) observed high DFS rates in early-stage cases, Rodriguez et al. (2021) noted that laparoscopic radical hysterectomy had inferior DFS compared to laparotomy (88.7% vs. 93.0%) [25,29]. Lee et al. (2011) found no significant difference in 5-year DFS between LRH and RAH [44].

Recurrence rates varied across surgical techniques. Rodriguez et al. (2021) reported a higher recurrence rate of 7.1% in the laparoscopic group compared to other approaches [29]. Kondo et al. (2022) found comparable recurrence rates of 4.7% in TLRH and 6.6% in O-RH [22]. Lee et al. (2011) observed slightly higher recurrence rates in RAH compared to LRH [44]. These findings suggest that while MIS techniques may offer comparable survival outcomes to open surgery, they may carry a higher risk of recurrence, particularly with laparoscopic surgery (Table 1).

- Operative time, blood loss, discharge time, and complications

Kondo et al. (2022) reported that TLRHs had shorter operative times and less blood loss than O-RHs, while Obermair et al. (2020) found MISs to have longer operative times but lower EBL compared to open surgeries [15,22]. Lee et al. (2011) noted that LRHs had longer operative times but significantly less blood loss than RAHs [44]. Discharge times were shorter for MISs in studies by Hayek et al. (2022) and Obermair et al. (2020), with LRHs also showing shorter hospital stays than RAHs [15,24,44]. Overall, these studies emphasize the benefits of MIS, such as reduced blood loss and shorter hospital stays, but they also highlight the increased recurrence risk with some MIS techniques, particularly laparoscopy, in the surgical treatment of cervical cancer (Table 3).

## 4. Discussion

The advancements in minimally invasive surgical techniques for gynecological cancers have substantially transformed the landscape of surgical management, particularly for endometrial and cervical cancers. Our findings align with those of previous studies, confirming that minimally invasive techniques not only ensure oncological safety but also improve perioperative outcomes.

Our evaluation of OS and DFS revealed that minimally invasive techniques are oncologically safe and often lead to outcomes comparable to traditional open surgeries. For example, the LAP2 study by Walker et al. (2012) [47] demonstrated no significant difference in 5-year OS between laparoscopies and laparotomies in women with stage I-IIA endometrial cancer, while also highlighting the benefits of shorter hospital stays and fewer complications associated with the laparoscopy. However, the results from the LACC trial by Ramirez et al. (2018) [48] challenge the oncological safety of minimally invasive surgery (MIS) in early-stage cervical cancer. The study found significantly lower DFS and OS rates for patients undergoing MISs compared to open radical hysterectomies, with a hazard ratio of 6.00 for OS. Additionally, the locoregional recurrence rates were higher in the MIS group, prompting the recommendation that open surgery should remain the standard of care for early-stage cervical cancer.

These concerns were further substantiated by the updated analysis from Ramirez et al. (2024) [49], which reported lower DFS (85.0% for MIS vs. 96.0% for open) and OS (90.6% for MIS vs. 96.2% for open) at the 4.5-year follow-up. The study highlighted a significantly higher recurrence rate for MIS (4.9%) compared to open surgery (1.8%), reinforcing the view that the open radical hysterectomy should be the preferred approach, particularly for high-risk early-stage cervical cancer patients.

Reijntjes et al. (2022) [13] found that a TLH without a lymphadenectomy is a safe and effective primary treatment for early-stage, low-grade endometrial cancer, reinforcing the notion that minimally invasive approaches can yield satisfactory survival rates. Gueli Alletti et al. (2021) [14] similarly demonstrated that both laparoscopic and robotic procedures provide oncological safety with outcomes comparable to open surgeries, supporting our findings that minimally invasive approaches can be effectively utilized in treating various stages of gynecological cancers.

However, some studies suggest nuanced differences in survival outcomes based on the type of hysterectomy performed. For instance, Shuai et al. (2024) [20] indicated that radical hysterectomy does not offer superior outcomes compared to total hysterectomy in stage II endometrial cancer. This underscores the importance of selecting appropriate surgical procedures tailored to individual patient needs rather than relying solely on aggressive surgical techniques. Yuk et al. (2021) [30] further highlighted improved survival outcomes for women with unsuspected uterine malignancies undergoing laparoscopic surgery, emphasizing the potential of minimally invasive strategies in achieving favorable oncological results.

Our findings regarding perioperative outcomes are consistent with those of Gaia et al. (2010), who found similar perioperative clinical outcomes between robotic and laparoscopic hysterectomies for endometrial cancer, with less blood loss but longer operative times for robotic cases [50].

Our analysis also demonstrated that minimally invasive surgeries typically result in shorter hospital stays and reduced EBL. Rambow et al. (2024) [21] reported that laparoscopic surgery for endometrial cancer maintains oncological safety and significantly reduces the duration of hospital stays. This finding is particularly relevant in today’s healthcare environment, where shorter hospital stays contribute to enhanced patient satisfaction and reduced healthcare costs.

The findings of Kondo et al. (2022) [22] align with our observation that TLH is effective with reduced bleeding and no significant difference in prognosis compared to open radical hysterectomy. This affirms that minimally invasive techniques can reduce perioperative morbidity without compromising surgical effectiveness. Furthermore, Hayek et al. (2022) [24] demonstrated that MIS offers similar oncological outcomes to open surgery while providing the additional benefits of shorter hospital stays and fewer complications, which aligns with our findings suggesting improved recovery profiles associated with MIS.

In terms of complications, our findings indicate that minimally invasive techniques tend to be associated with fewer postoperative complications. Renaud et al. (2022) [27] concluded that minimally invasive approaches, particularly robotic-assisted surgeries, are safe and effective, providing better outcomes, especially in elderly patients and those with early-stage, low-risk disease. This is significant as it suggests that robotic-assisted surgery could be a preferred option for vulnerable patient populations who may be at higher risk for postoperative complications. Additionally, Iavazzo et al. (2013) [51] suggested that the use of uterine manipulators in MIS does not correlate with the recurrence of endometrial carcinoma, indicating the procedure’s safety in such cases.

However, some studies have reported contrasting findings. Rodriguez et al. (2021) [29] noted that laparoscopic approaches were associated with worse DFS compared to laparotomies, indicating that the choice of surgical technique may influence long-term outcomes differently. This highlights the necessity for a comprehensive assessment of the patient’s condition and the potential risks involved in selecting surgical techniques. Nonetheless, the consensus from our review and the literature indicates that robotic and laparoscopic surgeries provide comparable, if not superior, complication profiles when contrasted with open surgeries.

The learning curve associated with robotic-assisted surgeries appears to influence surgical outcomes positively. Yotsumoto et al. (2022) [23] found that surgical performance in a robotic-assisted hysterectomy improves with experience, leading to better outcomes. This observation is essential for surgical teams considering adopting robotic techniques, as it emphasizes the need for ongoing training and skill development to maximize the benefits of robotic surgery. Lim et al. (2010) [45] further indicated that the learning curve for a robotic-assisted hysterectomy is generally easier than that for a laparoscopic hysterectomy, which could facilitate the transition to robotic techniques in surgical practice.

In comparison, a previous systematic review by Gaia et al. (2010) [50] noted that while robotic-assisted surgeries may require longer operative times initially, the outcomes improve significantly with experience. They further highlighted that robotic surgery offers reduced blood loss, fewer postoperative complications, and shorter hospital stays compared to open surgery. This aligns with our findings regarding the advantages of minimally invasive approaches. Moreover, Janda et al. (2017) [17] found that total abdominal hysterectomy and TLH resulted in an equivalent DFS at 4.5 years, reinforcing our observations that minimally invasive techniques do not compromise long-term oncological outcomes.

Overall, our findings and those of previous systematic reviews underscore the growing role of minimally invasive techniques in gynecological cancer surgery. These approaches offer comparable, if not superior, outcomes in terms of survival, perioperative safety, and complications when contrasted with traditional open surgeries.

## 5. Strength and Limitations

One of the key strengths of our study is its broader scope and detailed focus on clinical outcomes, distinguishing it from previous reviews like Scarpelli et al.’s. Our systematic review evaluates and compares the effectiveness of various surgical approaches, including OS, DFS, and recurrence rates, which are critical for assessing long-term outcomes [52]. By incorporating data from diverse studies, including RCTs and cohort studies from multiple countries, our review provides a global perspective on surgical effectiveness. This approach enhances the robustness of our findings and offers clinicians a nuanced understanding of the benefits and risks associated with each surgical method, guiding more informed decision-making in gynecological cancer treatment. The reviewed studies share limitations, such as variations in study design, sample sizes, and surgical technique definitions, which may affect generalizability. Future research should focus on standardized methodologies, larger multicenter trials, and long-term outcomes to improve the understanding of quality of life and survivorship in these patients.

## 6. Conclusions

Our systematic review supports the efficacy and safety of minimally invasive techniques in gynecological cancer surgery. The evidence indicates that robotic-assisted and laparoscopic surgeries provide comparable oncological outcomes, including overall survival (OS) and disease-free survival (DFS), to traditional open approaches across various gynecological cancers. Additionally, minimally invasive techniques generally show lower recurrence rates in endometrial and uterine cancers, although some studies report a higher recurrence risk for laparoscopic radical hysterectomies in early-stage cervical cancer. Alongside these oncological benefits, minimally invasive surgeries are associated with reduced blood loss, shorter hospital stays, and lower complication rates, enhancing perioperative recovery. These findings align with the results of previous studies, reinforcing the notion that minimally invasive techniques are increasingly becoming the preferred choice in gynecological oncology. Future research should continue to evaluate long-term outcomes and the cost-effectiveness of these surgical approaches to further guide clinical decision-making in this rapidly evolving field.

## Figures and Tables

**Table 1:** Demographic characteristics of surgical approaches for gynecological cancer.

Study ID	Country	Study Design	Population Criteria	Sample Size	Age (Mean ± SD) or Median(IQR)	Type of Cancer	Type of Invasive Surgery
Reijntjes B et al., 2022 [13]	Netherlands	RCT	Women with stage I endometrial cancer underwent TLH and TAH, without routine lymphadenectomy	Total: 263 (TLH: *n* = 175, TAH: *n* = 88)	TLH 62(48–89) TAH 64(39–86)	Stage I endometrial cancer	TLH and TAH, without lymphadenectomy
Gueli Alletti S et al., 2021 [14]	Italy	RCT	Patients undergoing minimally invasive staging for early-stage endometrial cancer	15,400	61 (31–81)	G1-G2 early-stage endometrial cancer	Laparoscopy (*n* = 93, 60.4%) and Robotic (*n* = 60, 39.0%)
Shuai X et al., 2024 [20]	China	Retrospective cohort	Women with stage II endometrial cancer who underwent hysterectomy, bilateral salpingectomy, bilateral oophorectomy, and pelvic lymphadenectomy	Total: 684 (Laparoscopy: *n* = 407, Laparotomy: *n* = 277)	57.9 ± 7.62 (minimally invasive: 57.5 ± 7.80)	Stage II endometrial cancer	Laparoscopy and Laparotomy
Rambow AC et al., 2024 [21]	Germany	Retrospective cohort	Women diagnosed with endometrial cancer	30,300	66.6 (38–90)	Stage Ia and Ib (77%), II (8.5%), III (12.3%), IV (1.7%)	TLH, TAH
Kondo E et al., 2022 [22]	Japan	Retrospective cohort	Women with early cervical cancer stage IB1-B2	9400	20.9 (15.1–35.4)	Stage IB1-B2 cervical cancer	Open-abdominal radical hysterectomy (O-RH) and total laparoscopic hysterectomy (TLRH)
Yotsumoto F et al., 2022 [23]	Japan	Retrospective cohort	Women with gynecologic tumors	3300	-	Gynecologic tumors	Robot-assisted hysterectomy (RAH) using da Vinci Xi surgical system
Hayek J et al., 2022 [24]	USA	Retrospective cohort	Women with stage IA1/IA2 cervical cancer	6230 (4054 underwent minimally invasive surgery)	Open: 46.3, MIS: 45.5	Stage IA1/IA2 cervical cancer	Hysterectomy (open or minimally invasive: laparoscopic or robotic-assisted)
Wojdat R et al., 2022 [25]	Germany	Retrospective cohort	Women with cervical cancer (stage I-IV)	124 (116 analyzed)	51.84 ± 15.41	Cervical cancer (stage I-IV)	Vaginal Assisted Radical Laparoscopic Hysterectomy (VARLH) (minimally invasive surgery)
Segarra-Vidal B et al., 2021 [26]	Not Mentioned	Retrospective cohort	Patients with Grade 3 endometrial cancer	626 (MIS = 263(42%) Open surgery = 363 (58%)		Grade 3 endometrial cancer	Hysterectomy, bilateral salpingo-oophorectomy, and staging
Renaud MC et al., 2022 [27]	Canada	Retrospective cohort	Patients with endometrial cancer surgeries	Total: 735 (Laparotomy: *n* = 254, MIS: *n* = 461)	LAP: 65.4 (40–92); LAVH: 66.6 (36–82); TLH: 63.0 (41–87); RAH: 63.4 (30–90); Total MIS: 63.8 (30–90)	Endometrial cancer	Laparotomy, robotic-assisted hysterectomy, TLH, LAVH
Nasioudis D et al., 2021 [28]	USA	Retrospective cohort	Patients with stage IA cervical carcinoma undergoing hysterectomy	1930 (Open: *n* = 685, Laparoscopic: *n* = 438, Robotic: *n* = 807)	43 (22–87)	Stage IA cervical carcinoma	Simple hysterectomy, radical hysterectomy
Rodriguez J et al., 2021 [29]	Colombia, USA, Mexico, Italy, Peru, Spain	Retrospective cohort	Patients diagnosed with early-stage cervical cancer who underwent radical hysterectomy and pelvic lymphadenectomy	1379 (Laparoscopic: *n* = 681, Laparotomy: *n* = 698)	-	Early-stage cervical cancer (IA1, IA2, IB1)	Radical hysterectomy and pelvic lymphadenectomy, either by minimally invasive surgery or open approach
Yuk JS et al., 2021 [30]	Republic of Korea	Retrospective cohort	Women with unsuspected uterine malignancy diagnosed after laparotomic versus laparoscopic hysterectomy	157,232 (Laparotomic: *n* = 103,631, Laparoscopic: *n* = 53,601)	46.29 ± 0.02	Unsuspected endometrial cancer	Laparotomic and laparoscopic hysterectomy
Mereu L et al., 2020 [31]	Italy	Case–control	Patients with a biopsy-confirmed diagnosis of low-risk endometrial cancer or atypical endometrial hyperplasia	76 (Robotic multiport: *n* = 51, Robotic single-site: *n* = 25)	61.7 ± 11.0	Grade 1–2 endometrial cancer	Robotic total laparoscopic hysterectomy and sentinel lymph node mapping
Obermair A et al., 2020 [15]	24 countries	RCT	Women with stage IA1-IB1 cervical cancer who underwent minimally invasive or open radical hysterectomy	636 (536 for analysis, Minimally Invasive: *n* = 279, Open: *n* = 257)	4600	Stage IA1-IB1 cervical cancer	Minimally invasive surgery, open radical hysterectomy
Jørgensen SL et al., 2019 [32]	Denmark	Prospective cohort	Women with early-stage endometrial cancer who underwent surgery before and after RMIS introduction	3091 (before RMIS), 2563 (after RMIS)	Group 1: 66 (29–96), Group 2: 67 (33–98)	Early stage endometrial cancer	Laparoscopic minimally invasive surgery, robotic minimally invasive surgery, total abdominal hysterectomy
Corrado G et al., 2018 [33]	Italy	Retrospective cohort	Obese patients (BMI > 30 kg/m^2^) with primary, histologically confirmed endometrial carcinoma	655 (Robotic: *n* = 249, Laparoscopic: *n* = 406)	Robotic G1: 64 (39–87), G2: 63 (35–80), G3: 59 (30–78), G4: 57.5 (40–70)	Endometrial cancer	Laparoscopic hysterectomy, robotic hysterectomy
Ruan XC et al., 2018 [34]	Singapore	Retrospective cohort	Women with surgical Stage 1 endometrioid adenocarcinoma of the uterus	374 (Laparotomy: *n* = 229, Laparoscopy: *n* = 145)	Laparotomy: 55.6 ± 9.7, Laparoscopy: 53.0 ± 11.0	Stage 1A and 1B endometrial cancer	Laparotomy, laparoscopy
Versluis MAC et al., 2018 [35]	Netherlands	Retrospective cohort	Women with uterine carcinosarcoma (UCS)	1140 (TAH-BSO: *n* = 893, TAH-BSO + LND: *n* = 247)	70 (IQR 62–77)	Uterine carcinosarcoma (UCS)	Hysterectomy, bilateral salpingo-oophorectomy, and lymphadenectomy (LND)
Lee J et al., 2018 [36]	USA	Retrospective cohort	Women who underwent hysterectomy for endometrial cancer	17,692 (Abdominal: *n* = 5496, Laparoscopic: *n* = 11,878, Vaginal: *n* = 318)	White: 63 ± 11, Black: 63 ± 10	Endometrial cancer	Laparoscopic hysterectomy, abdominal hysterectomies, vaginal hysterectomies
Higgs P et al., 2018 [16]	Australia	RCT	Women who underwent surgical treatment for early-stage endometrial cancer.	760	-	Endometrial cancer	Total abdominal hysterectomy versus total laparoscopic hysterectomy
Eisenkop SM et al., 2018 [37]	USA	Retrospective case–control study	Patients with endometrial cancer	297	63.2 (32.4–90.9 years)	Endometrial cancer	Total laparoscopic hysterectomy with bilateral salpingoophorectomy and pelvic/aortic lymph node dissection (TLH/BSO/LND)
Torng PL et al., 2017 [38]	Taiwan	Retrospective cohort	Patients with endometrial cancer who underwent laparoscopic (LSS) or robotic-assisted staging surgery (RSS)	Total 44 (24 LSS, 20 RSS)	56.9 ± 7.1	Endometrial cancer	Laparoscopic (LSS) or robotic-assisted staging surgery (RSS)
Moukarzel LA et al., 2017 [39]	USA	Retrospective cohort	Patients with endometrial cancer who underwent R-LESS TLH with SLN mapping and MPR TLH with SLN mapping	Total 27 (14 R-LESS, 13 MPR)	-	Endometrial cancer grade 1/2	Robotic laparoendoscopic single-site (R-LESS); multiport robotic (MPR) total laparoscopic hysterectomy (TLH)
Janda M et al., 2017 [17]	Australia, New Zealand, and Hong Kong	RCT	Women who underwent laparoscopic hysterectomy (TLH) and total abdominal hysterectomy (TAH)	Total 760 (353 TAH, 407 TLH)	TAH: 63.1 (10.6), TLH: 63.3 (10.0)	Stage 1 endometrial cancer	Total laparoscopic hysterectomy (TLH) is equivalent to total abdominal hysterectomy (TAH)
Zakhari A et al., 2016 [40]	Canada	Retrospective cohort	Women 80 years old or more undergoing robotic and laparoscopic hysterectomy for uterine cancer	915	80–84: 57.7, 85–90: 34.61, 90+: 7.63	Uterine cancer	Robotic and laparoscopic hysterectomy
Corrado G et al., 2016 [41]	Italy	Case–control study	Women with stage 1A/B endometrial cancer who underwent robotic single-site hysterectomy (RSSH) versus robotic multiport hysterectomy (RMPH)	Total 69 (23 RSSH, 46 RMPH)	RSSH: 64 (35–85), RMPH: 59 (38–88)	Stage 1A/B endometrial cancer	Robotic hysterectomy, robotic single-site hysterectomy, robotic multiport hysterectomy
Somashekhar SP et al., 2014 [18]	India	RCT	Patients with unexpected uterine sarcoma incidentally diagnosed after total laparoscopic or abdominal hysterectomy	Total 50 (25 robotic, 25 open)	Robotic: 51.44; Open: 53	Uterine cancer	Robotic-assisted hysterectomy, Lymphadenectomy
Malinowski A et al., 2013 [42]	Not Mentioned	Retrospective cohort	Patients with endometrial cancer who underwent total laparoscopic hysterectomy (TLH) and laparotomy	Total 63 (31 TLH, 42 laprotomy)	TLH: 54, Laprotomy: 64	Endometrial cancer	Total laparoscopic hysterectomy (TLH), laparotomy
Lajer H et al., 2012 [43]	Denmark	Prospective cohort	Patients with stage 1A endometrial cancer who underwent abdominal hysterectomy and bilateral salpingo-oophorectomy without adjuvant therapy	571	62.9 (28.3–87.0 years)	Stage 1A endometrial cancer	Abdominal hysterectomy, bilateral salpingo-oophorectomy
Lee EJ et al., 2011 [44]	Republic of Korea	Case–control study	Patients with stage I–II cervical cancer who underwent laparoscopic radical hysterectomy (LRH) and radical abdominal hysterectomy (RAH)	Total 72 (24 LRH, 48 RAH)	LRH: 48.4 (39–68), RAH: 50.2 (34–67)	Stage I-II cervical cancer	Laparoscopic radical hysterectomy (LRH) and radical abdominal hysterectomy (RAH)
Lim PC et al., 2010 [45]	USA	Case–control study	Patients with endometrial cancer who underwent robotic-assisted hysterectomy with bilateral lymphadenectomy, laparoscopic hysterectomy with bilateral lymphadenectomy, and traditional total abdominal hysterectomy with lymphadenectomy	Total 148 (R: 56, L: 56, T: 36)	R: 62.5 (8.4), L: 61.4 (11.7), T: 62.7 (10.6)	Endometrial cancer	Robotic-assisted hysterectomy with bilateral lymphadenectomy, laparoscopic hysterectomy with bilateral lymphadenectomy, traditional total abdominal hysterectomy with lymphadenectomy
Devaja O et al., 2010 [46]	UK	Prospective cohort	Patients with endometrial cancer who underwent Laparoscopically Assisted Vaginal Hysterectomy (LAVH) and total abdominal hysterectomy (TAH)	Total 182 (LAVH: 70, TAH: 112)	LAVH: 65.84 (10.512), TAH: 64.99 (11.042)	Endometrial cancer	Laparoscopically Assisted Vaginal Hysterectomy (LAVH) and total abdominal hysterectomy (TAH)
Janda M et al., 2010 [19]	Australia, New Zealand, and Hong Kong	RCT	Patients with stage I endometrial cancer who underwent total laparoscopic hysterectomy (TLH) and total abdominal hysterectomy (TAH)	Total 332 (TLH: 190, TAH: 142)	TLH: 62.8 (10.0), TAH: 62.7 (9.7)	Stage 1 endometrial cancer	Total laparoscopic hysterectomy (TLH) and total abdominal hysterectomy (TAH)

TLH: total laparoscopic hysterectomy; TAH: total abdominal hysterectomy; MIS: minimally invasive surgery, which can include laparoscopic or robotic-assisted techniques; RH: robotic hysterectomy; RAH: robotic-assisted hysterectomy; LND: lymph node dissection; O-RH: open radical hysterectomy; TLRH: Total Laparoscopic Radical Hysterectomy; VARLH: Vaginal Assisted Radical Laparoscopic Hysterectomy; LAVH: Laparoscopically Assisted Vaginal Hysterectomy; R-LESS: Robotic Laparoendoscopic Single-Site Surgery; MPR: Multiport Robotic Surgery; SLN Mapping: sentinel lymph node mapping; BSO: bilateral salpingo-oophorectomy; RCT: randomized controlled trial; RMIS: robotic minimally invasive surgery; IQR: Interquartile Range.

**Table 2:** Comparison of surgical outcomes in laparoscopic, robotic-assisted, and open hysterectomy for gynecological cancers.

Study ID	OS	DFS	Recurrence	Follow-Up	Key Findings
Reijntjes B et al., 2022 [13]	TLH vs. TAH: 89.2% vs. 82.8% (5 years)	TLH vs. TAH: 90.3% vs. 84.1% (5 years)	aHR (recurrence): 0.69 (95% CI, 0.31–1.52)	5 years	Comparable disease recurrence and 5-year survival rates between TLH and TAH. TLH is effective for early-stage, low-grade endometrial cancer without lymphadenectomy.
Gueli Alletti S et al., 2021 [14]	Not reached	Not reached	Not reached	38.7 months (95% CI 37.1–40.8)	Significant difference in OT for laparoscopic staging, no difference in survival or recurrence between groups.
Shuai X et al., 2024 [20]	Laparotomy vs. Laparoscopy: 85.5% vs. 82.7% (HR = 1.00, *p* = 0.99)	Laparotomy vs. Laparoscopy: 88.7% vs. 87.1% (HR = 1.22, *p* = 0.34)	98 (14.3%) total recurrences	77 months	No significant difference in 5-year DFS and OS between laparotomy and laparoscopy, or between radical and simple hysterectomy.
Rambow AC et al., 2024 [21]	TAH: 84.9%, TLH: 85.3% (*p* = 0.85)	-	-	5 years	TLH resulted in shorter postoperative stays without affecting OS.
Kondo E et al., 2022 [22]	TLRH: 97.2%, O-RH: 100%	TLRH: 92.6%, O-RH: 92.6%	TLRH: 4.7%, O-RH: 6.6%	33.5 months (TLRH), 41.5 months (O-RH)	No significant difference in PFS and OS between TLRH and O-RH. TLRH had less bleeding.
Hayek J et al., 2022 [24]	Open: HR 1.23 (CI 0.92–1.63)	-	-	-	MIS had shorter hospital stays and fewer complications, with no significant difference in survival.
Wojdat R et al., 2022 [25]	Stage IA: 151.23 months, Stage IB2-IV: 105.56 months	-	None	45.6 months	High 3- and 5-year DFS and OS rates with no recurrence in early-stage cervical cancer.
Segarra-Vidal B et al., 2021 [26]	HR 1.04 (95% CI 0.73–1.48, *p* = 0.81)	5-year OS: Open: 53.4%, MIS: 54.6%	HR 0.99 (95% CI 0.69–1.44, *p* = 0.99)	5 years	No significant difference in DFS, OS, or recurrence between open and MIS in high-risk endometrial cancer.
Renaud MC et al., 2022 [27]	*p* = 0.02	-	Higher in LAP vs. MIS (*p* < 0.05)	LAP: 45.4 months, LAVH: 58.8 months	Robotic-assisted hysterectomy had fewer complications and shorter hospital stays, with a survival benefit in elderly patients.
Nasioudis D et al., 2021 [28]	MIS: 21 deaths, Open: 16 deaths (*p* = 0.87)	-	-	38.1 months	MIS resulted in shorter hospital stays with no difference in OS compared to open surgery.
Rodriguez J et al., 2021 [29]	4-year DFS: 88.7% (laparoscopy) vs. 93.0% (laparotomy)	-	Laparoscopy: 7.1% recurrence	52.1 months	Laparoscopic radical hysterectomy had inferior DFS compared to laparotomy.
Yuk JS et al., 2021 [30]	Laparoscopy group had higher OS than laparotomy (*p* < 0.001)	-	-	7 years	Laparoscopy showed a favorable OS for unsuspected uterine malignancies.
Jørgensen SL et al., 2019 [32]	Group 1: HR 1.22, Group 2: HR 1.42	-	-	8.8 years (Group 1), 4.4 years (Group 2)	Robotic surgery improved OS compared to traditional approaches.
Corrado G et al., 2018 [33]	3.7% deaths (LH), 2.8% deaths (RH), OS: 0.791	DFS: *p* = 0.869	34 (LH), 17 (RH)	33 months (LH), 26.7 months (RH)	Robotic surgery had longer OT but reduced hospital stays, with comparable outcomes across BMI variations.
Ruan XC et al., 2018 [34]	-	Laparoscopy: 11.8 months, Laparotomy: 8 months	Laparoscopy: 4, Laparotomy: 4	-	Laparoscopic surgery had reduced pain, shorter stays, and less blood loss.
Versluis MAC et al., 2018 [35]	OS: 2.03 years	DFS: 1.53 years	-	-	Lymph node dissection (>10 nodes) improved OS.

OS: overall survival; DFS: disease-free survival; Recurrence: recurrence rate; aHR: Adjusted Hazard Ratio; CI: Confidence Interval; TLH: total laparoscopic hysterectomy, TAH: total abdominal hysterectomy; TLRH: Total Laparoscopic Radical Hysterectomy; O-RH: open radical hysterectomy; PFS: Progression-Free Survival; MIS: minimally invasive surgery; LAP: laparoscopy; LAVH: laparoscopic-assisted vaginal hysterectomy; OT: operative time; LH: laparoscopic hysterectomy; RH: robotic hysterectomy; BMI: Body Mass Index.

**Table 3:** Operative characteristics, blood loss, discharge time, and complications of various surgical studies.

Study ID	Operative Time (Min)	Estimated Blood Loss (EBL, mL)	Discharge Time (Days)	Complications
Gueli Alletti S et al., 2021 [14]	Laparoscopy: 150 (50–370), Robotic: 170 (50–300)	Laparoscopy: 50 (0–550) Robotic: 50 (0–250)	2 (1–3)	5 (3.2% intraoperative), 4.6% early postoperative
Kondo E et al., 2022 [22]	TLRH 382 (270–460) O-RH 382 (200–689)	417	-	-
Hayek J et al., 2022 [24]	-	-	1.35 (MIS) 3.08 (open)	-
Renaud MC et al., 2022 [27]	LAP: 137, LAVH: 182, TLH: 197, RAH: 164	LAP: 300, LAVH: 140, TLH: 111, RAH: 93	LAP: Day 0; LAVH: Day 11; TLH: Day 49; RAH: Day 178	(<30 d; *p* = 0.0002)
Mereu L et al., 2020 [31]	154.8	<100 mL: 88.2% >100 mL: 11.8%	2.8	Intra-op: 3.9%, Post-op: 5.2%
Obermair A et al., 2020 [15]	MIS: 216, Open: 187	MIS: 101, Open: 209	MIS: 3 days, Open: 5 days	-
Corrado G et al., 2018 [33]	LH: 131.9, RH: 183.5	LH: 85.1, RH: 124.1	LH: 3.6, RH: 3.1	-
Ruan XC et al., 2018 [34]	Laparoscopy: 178.5, Laparotomy: 187.2	Laparoscopy: 155.5 Laparotomy: 78.6	Laparoscopy: 7 Laparotomy: 4.7	-

TLRH: Total Laparoscopic Radical Hysterectomy; O-RH: open radical hysterectomy; MIS: minimally invasive surgery; LAP: Laparoscopy; LAVH: laparoscopic-assisted vaginal hysterectomy; TLH: total laparoscopic hysterectomy; RAH: robotic-assisted hysterectomy; LH: laparoscopic hysterectomy; RH: robotic hysterectomy; EBL: estimated blood loss; Intra-op: intraoperative; Post-op: postoperative; min: Minutes; ml: Milliliters; d: days.

## Data Availability

The data presented in this study are available on request from the corresponding author. Access may be subject to certain restrictions.
